# Small‐Molecule Kinase Inhibitors Modulating Circadian Rhythms

**DOI:** 10.1002/med.70071

**Published:** 2026-06-15

**Authors:** Irene Castellino, Manuela Bartolini, Rudolf Andrys, Kamil Musilek

**Affiliations:** ^1^ Department of Chemistry, Faculty of Science University of Hradec Kralove Hradec Kralove Czech Republic; ^2^ Department of Pharmacy and Biotechnology Alma Mater Studiorum University of Bologna Bologna Italy

**Keywords:** circadian clock, circadian rhythm, inhibitor, kinase

## Abstract

The circadian clock mechanism generates 24‐h rhythms crucial for regulating various physiological processes, and its dysregulation has been implicated in numerous diseases. In cells, the circadian clock operates through a transcriptional‐translational feedback loop, where phosphorylation plays a pivotal role in maintaining accurate circadian rhythms. Consequently, kinase inhibitors have emerged as promising targets for modulating the circadian clock and potentially treating circadian‐related diseases. This review aims to provide an overview of the current state‐of‐the‐art of kinase inhibitors with effects on the mammalian circadian clock. By highlighting promising targets and addressing the limitations of existing inhibitors, this review intends to provide a guide for future research efforts towards the development of novel compounds for the treatment of circadian‐related disorders. Furthermore, it highlights the critical discrepancy between *in vitro* activity and *in vivo* effectiveness, emphasizing the critical need for rigorous *in vivo* validation to translate the therapeutic potential of kinase inhibitors into effective treatments for these disorders.

## 1 Introduction

The circadian rhythm is a complex biological mechanism that creates a daily rhythm with a period of approximately 24 h. In mammals, nearly all cells possess the capacity to generate these oscillations, creating a hierarchical system composed of a central pacemaker in the suprachiasmatic nucleus (SCN) of the hypothalamus and the peripheral clocks found in most tissues and organs. This master clock primarily regulates general functions such as mood and behavior, while peripheral clocks manage tissue‐specific processes like hormone secretion, metabolism, heart rate, and body temperature. The SCN coordinates these cellular oscillators throughout the body via neural and hormonal signals, ensuring that various physiological functions remain synchronized with the external environment [1]. The disruption of the circadian clock (CC), by environmental or genetic factors, is increasingly recognized as contributor tothe onset and progression of various diseases. Consequently, the identification of molecules capable of stabilizing and/or restoring the CC, potentially alleviating metabolic disorders, cancer, inflammation, sleep, and mood disorders, has attracted significant attention [2].

### 1.1 The Molecular Architecture of the Circadian Clock and the Central Role of Kinases

Peripheral clocks and SCN neurons share the same molecular architecture, but the SCN neurons possess a more robust network [3]. The molecular mechanism of the CC in mammals is generated by cell‐autonomous transcriptional‐translational feedback loops (TTFLs), and includes a core loop and a secondary stabilization loop that work together to produce and maintain robust 24 h oscillations of gene transcription (schematically represented in Figure 1). Furthermore, a complex network of post‐transcriptional modifications, including phosphorylation, ubiquitylation, and acetylation, plays a crucial role in orchestrating essential processes like nuclear entry, protein complex formation, and protein degradation of the clock factors involved in the TFFLs. These modifications act as fine‐tuning mechanisms, ensuring the precise function of the circadian rhythm [4]. Among these post‐transcriptional modifications of CC, phosphorylation plays a key role; hence, the involved kinases have undergone a drastic increase in interest in the last decade [5, 6]. Despite this growing interest, the complex network of kinase‐mediated regulation of the CC remains only partially elucidated. Accordingly, the identification and characterization of these regulatory kinases together with their respective small‐molecule modulators, thus, represent a promising area of research.

**Figure 1:**
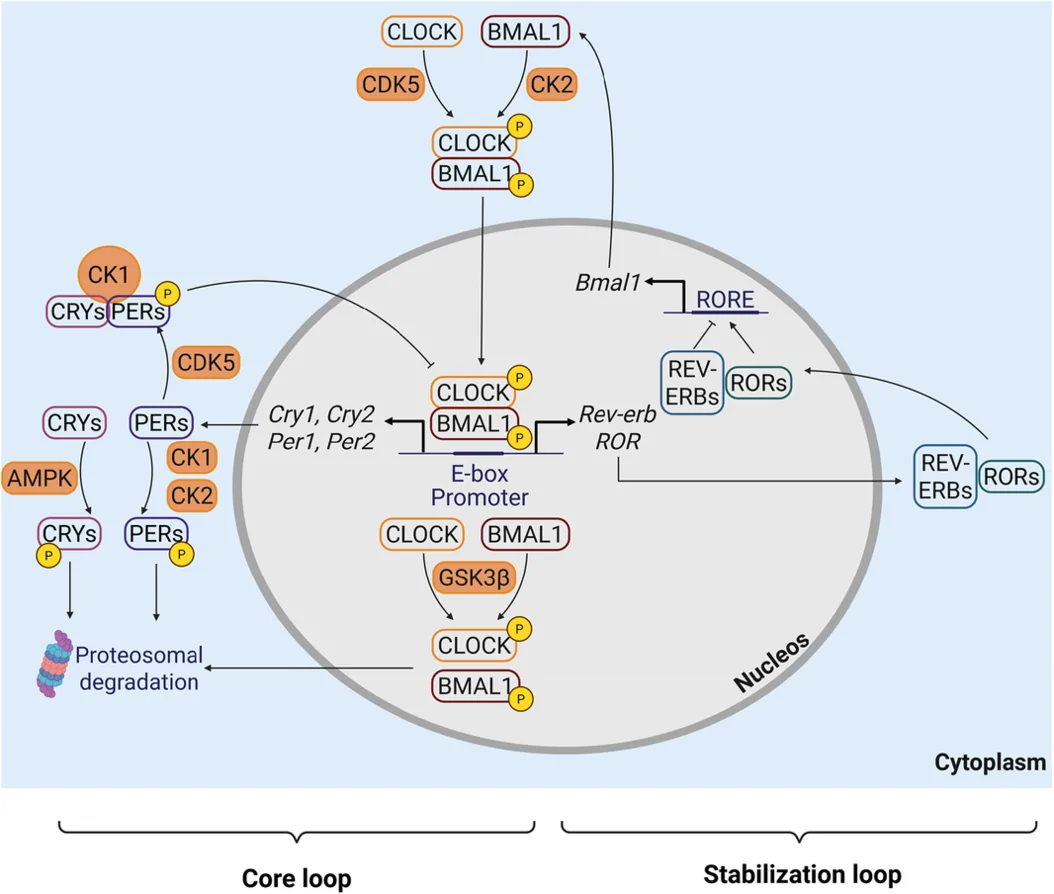
Schematic representation of the mammalian cellular circadian clock, highlighting the role of key kinases in the TTFLs. In the core feedback loop, the activators CLOCK and BMAL1 heterodimerize in the cytosol, a process facilitated by phosphorylation via CDK5 and CK2, respectively. The resultant CLOCK‐BMAL1 complex translocates in the nucleus and binds to E‐box domains on gene promoters, inducing the transcription of target genes, including *Per1*, *Per2*, *Cry1*, and *Cry2*. PER proteins, after initial phosphorylation by CDK5, dimerize with CRY proteins. PER‐CRY heterodimer associates with CK1 and translocate to the nucleus. PER‐CRY‐CK1 complex dissociates the CLOCK‐BMAL1 complex from the chromatin, thereby repressing their own transcription. Once relieved from chromatin, GSK3β targets CLOCK and BMAL1 for degradation via the proteasomal pathway. The negative regulation imposed by the PER‐CRY complex is relieved through their proteasomal degradation, controlled by various kinases, including AMPK, CK1, and CK2. In the stabilization loop, CLOCK‐BMAL1 induces the transcription of genes for REV‐ERBs and RORs. They compete at the RORE on the BMAL1 gene promoter, providing both positive (RORs) and negative (REV‐ERBs) regulation of BMAL1 transcription. Figure created with BioRender.com. [Color figure can be viewed at wileyonlinelibrary.com]

The leading actors of the TTFL are four clock proteins: two activators, Circadian Locomotor Output Cycles Kaput (CLOCK) and Brain and Muscle ARNT‐Like 1 (BMAL1), and two repressors, Period (PER) and Cryptochrome (CRY). In the core loop, the transcription factor CLOCK interacts with BMAL1 through the PAS (Per‐Arnt‐Sim) and bHLH (basic‐helix‐loop‐helix) domains, inducing dimerization. This dimerization is facilitated by phosphorylation, specifically by cyclin‐dependent kinase 5 (CDK5) and casein kinase 2 (CK2) on CLOCK and BMAL1, respectively [6, 7, 8]. The resulting heterodimer binds the E‐boxes DNA elements of the promoters of *Period1/2* and *Cryptochrome1/2* genes. Consequently, PER and CRY proteins are expressed. When their concentrations in the cytosol rise, PER and CRY proteins heterodimerize, interact with casein kinase 1δ (CK1δ) and ε (CK1ε), and translocate into the nucleus [9]. The formation of this complex, called “PER complex,” requires phosphorylation of PER factors by CDK5; although other required phosphorylation where identified the involved kinases remain to be fully clarified [10]. In the nucleus, the “PER‐complex” inhibits CLOCK‐BMAL1 in order to repress *Per* and *Cry* transcription [9]. When CLOCK and BMAL1 are separated from the chromatin they are phosphorylated by glycogen synthase kinase 3β (GSK3β), and other yet‐unknown kinases, which targets them for proteasome degradation in the cytoplasm [11, 12]. The negative regulation of the PER‐complex is relieved through degradation of PER and CRY proteins via the proteasome pathway, mediated by various kinases including AMPK, CK1, CK2 [5, 6]. Thanks to that, the repression of CLOCK‐BMAL1 is relieved, and the cycle begins again [13].

In addition to the core loop, CLOCK‐BMAL1 complex also binds the E‐box DNA elements of the gene promoters encoding for REV‐ERBs and RORs (Retinoic‐acid‐receptor‐related Orphan Receptors). These two elements compete at the same DNA‐binding element (RORE) in order to regulate BMAL1 transcription [1].

### 1.2 Circadian Disruption and Related Pathologies

Circadian disruption is used as a general term referring to any perturbation of circadian physiology caused by environmental factors, such as night work, jet lag or exposure to artificial light, or genetic factors, as mutations in clock genes. The term includes circadian misalignment (when two or more rhythms adopt an abnormal phase relationship) and circadian desynchronization (when two or more rhythms exhibit a different period or rate of oscillation). These perturbations can be internal, for example, between the central pacemaker and a peripheral clock, or external, for example, between an internal clock and the environmental light‐dark cycle [14]. Disruptions to the circadian rhythm are tightly linked to the incidence and progression of various diseases [15, 16].

Circadian disruption is strongly implicated in metabolic syndrome and cardiovascular disease. In shift workers and individuals experiencing social jetlag (discrepancy in sleep patterns between workdays and days off) this misalignment is associated with elevated BMI, increased insulin resistance, and impaired glucose control with increased risk for obesity, diabetes, hypertension, and dyslipidemia [17]. Circadian rhythm disruption is a key feature of sleep disorders and mood disorders, including anxiety, Major Depressive Disorder, and bipolar disorder [15]. The sleep disorder familial advanced sleep phase syndrome (FASPS) is associated with a mutation in the CK1δ/ε, leading to the hypophosphorylation of the PER2 protein and a desynchronization in the CC, with shortened circadian period [18]. Mood disorders, such as mania‐like behavior in *Clock* ^*Δ19/Δ19*^ mice, are linked to the absence of REV‐ERBα activity. The classic mood stabilizer Lithium targets the GSK‐3β kinase, which stabilizes REV‐ERBα, demonstrating a direct link between kinase activity, the clock, and mood regulation [19]. Circadian disruption appears to play a bidirectional role in neurodegenerative disorders, where dysregulation may both be a symptom and accelerate pathology [15, 20]. Parkinson's disease (PD) is characterized by a reduction in overall circadian amplitude. This may increase the risk for motor dysfunction by triggering a neuroinflammatory response and neurodegeneration of the nigro‐striatal pathway. Dysfunction in the dopamine system, a hallmark of PD, may further alter the circadian response to light and impact the gene expression Per2 [15]. In Alzheimer's disease (AD), the rest/activity rhythm progressively dampens in amplitude and becomes arrhythmic, correlating with disease severity. Evidence suggests that circadian disruption may accelerate disease progression by increasing tau deposition and neurodegeneration in vulnerable individuals. Furthermore, the circadian disruption seen in AD is associated with reduced physiological levels of melatonin, a compound that normally protects against Aβ‐amyloid aggregation and toxicity, thereby potentially accelerating disease progression [15, 20].

Given the profound impact of circadian misalignment on systemic health and disease progression, there is a compelling need for interventions to restore clock synchronization. Three major therapeutic strategies for circadian dysfunction are currently under investigation [21]:

− Training the Clock (behavioral and environmental interventions): this approach aims to maintain robust circadian rhythms by optimizing external cues, such as reducing evening exposure to blue light and increasing daytime light exposure, consolidating the eating period to a defined interval profoundly sustains robust circadian rhythms in peripheral organs, like the liver, improves metabolic health, and can impact brain health.
− Chronotherapy: this strategy focuses on optimize the timing of drug administration to increase efficacy and reduce adverse side effects by aligning the dose with the natural daily rhythm of the body or the drug target.
− Drugging the Clock: this approach involves the use of small‐molecule agents to directly target a component of the circadian clock or a closely associated regulator.

While behavioral and environmental training of the CC can positively impact systemic health, its efficacy is often limited in severe or chronic conditions, such as neurodegenerative disorders (NDs). Consequently, there is a growing necessity for pharmacological modulators that target the clock's core machinery. However, although several classes of small molecules have shown promise in preclinical models, their successful translation into clinical practice remains a significant challenge.

### 1.3 Strategies for Developing Circadian Clock Modulators

Approaches to developing new circadian‐clock modulators can be broadly divided into two main categories: target‐based and unbiased strategies (Figure 2) [22]. The most common unbiased screens rely on cell‐based assays that employ a luciferase reporter. These assays evaluate the transcriptional activity of the CLOCK‐BMAL1 complex by monitoring the expression of a luciferase reporter gene regulated by E‐box elements [23, 24]. In a mechanism‐based assay, a cell line with the luciferase reporter, commonly *Bmal1*‐d*Luc* and *Per1/2*‐d*Luc*, is treated with libraries of compounds, and the CLOCK‐BMAL1 transactivation is monitored by a determination of an enhanced or reduced bioluminescence signal [22, 25]. The same principle is used in the so‐called phenotypic assays. Unlike the previous method, the used cell lines possess a robust circadian rhythmicity after a synchronization treatment with dexamethasone, forskolin, or serum shock. Thus, the bioluminescence rhythm remains stable over several days. In this assay, it is possible to evaluate the effect of molecules on the CC by determining period length, phase, amplitude, and robustness of the oscillations [22, 26]. While unbiased screens can identify novel CC modulators, subsequent validation studies are necessary to determine their precise targets. These approaches offer the advantage of discovering unexpected targets and mechanisms that influence circadian rhythms.

**Figure 2:**
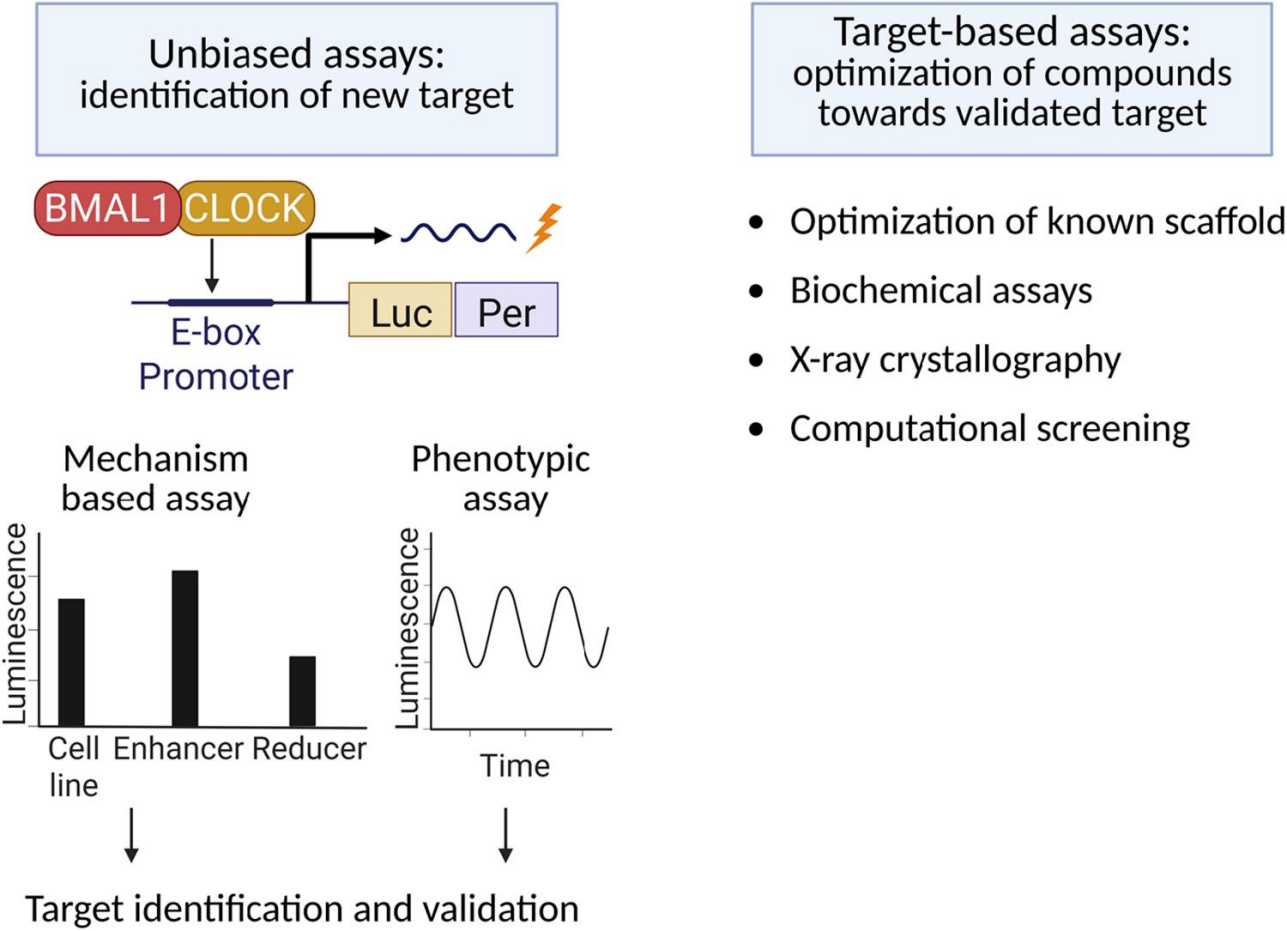
Approaches to developing CC modulators. The figure illustrates the two primary strategies used for developing new compounds that modulate the CC: unbiased assays (for identification of new CC targets) and target‐based assays (for compound optimization). Unbiased assays use a luciferase (*Luc*) reporter driven by an E‐box promoter to monitor the transcriptional activity of the BMAL1/CLOCK complex. In mechanism‐based assay, it is measured the immediate effect of a compound on the steady‐state luminescence (enhancer/reducer), while in phenotypic assay it is measured the compound's effect on the rhythmic properties (period, phase, amplitude) of the clock over time. The strategies used in target‐based assay aimed at the identification of compounds towards a validated CC target protein, including scaffold optimization, biochemical assays, X‐ray crystallography and computational screening. Figure created with BioRender.com. [Color figure can be viewed at wileyonlinelibrary.com]

The target‐based assays serve as alternatives to unbiased methods for developing new scaffolds or optimizing known molecules that target a validated CC protein. This approach involves several steps, including the molecular design of known scaffolds, computational screening of compound libraries towards a specific target, biochemical assays to confirm the interaction between the ligand and the clock protein, and/or the use of X‐ray crystallography to elucidate the precise mode of interaction. Although promising *in vitro* activity is a good starting point for developing circadian‐clock modulators, the complex interplay of factors characterizing living organisms makes *in vivo* evaluation in animal models a mandatory step. Indeed, the observed effects in whole organisms often differ significantly in magnitude from those found in *in vitro* assays [27, 28].

### 1.4 Kinases as Potential Molecular Targets

Numerous potential targets have been identified for modulating the CC, including core‐clock proteins and non‐core‐clock proteins [16, 29]. As our understanding of these pathways deepens, the number of potential targets also expands. However, to identify promising drug candidates, it is crucial to focus on molecular targets that are amenable to small‐molecule modulation and whose modulation can elicit a sufficient impact on the circadian period. In this scenario, kinases have emerged as promising targets for modulating the CC. Indeed, they can be effectively targeted with small‐molecule drugs and play a fundamental role in maintaining accurate circadian rhythms. In fact, phenotype‐based chemical screens have consistently identified kinase inhibitors as potent modulators of the CC, highlighting the significant impact of targeting these enzymes [30, 31, 32]. However, the involvement of kinases in various cellular pathways, along with their lack of selectivity, poses significant challenges for their use as therapeutic targets [33].

This review aims to provide an overview of the current state‐of‐the‐art of kinase inhibitors that can affect the CC. By highlighting promising targets and discussing the limitations of existing inhibitors, this review aims to provide a guide for future research efforts towards developing novel compounds for the treatment of circadian‐related disorders. While this review focuses on kinase inhibitors, it is also important to note that small‐molecule modulators targeting other core‐clock proteins have also been identified. For a comprehensive overview of modulators interfering with other CC proteins, readers may refer to dedicated reviews [16, 34, 35].

### 1.5 Casein Kinase 1 Family

CK1s are highly conserved serine/threonine kinases with a monomeric structure (Figure 5A), found across various eukaryotic organisms; in mammals, seven distinct CK1 isoforms exist (α, β, γ1, γ2, γ3, δ, and ε) [36, 37]. CK1δ and CK1ε stand out for their critical role in maintaining the 24‐h circadian rhythm and can be regarded as the master regulators of the CC. They accomplish this role by specifically targeting and phosphorylating the proteins PER1 and PER2 at multiple sites. Such phosphorylation regulates various cellular processes, including the movement of PER proteins into the nucleus, which is a critical step for their function. Furthermore, the phosphorylation of PER proteins at specific sites marks them for ubiquitination and subsequent proteasomal degradation [38, 39, 40]. As described above, CK1δ/ε interacts with PER and CRY proteins and forms the so‐called “PER‐complex” that acts as a brake on the CC by inhibiting the interaction of CLOCK‐BMAL1 with the E‐box promoters. Interestingly, CK1δ/ε remains attached to PER proteins throughout the entire circadian cycle [9]. The different effects of CK1δ/ε on PER factors are mediated by the phosphorylation of two specific sites located in the Degron and in the FASP regions (schematic representation in Figure 3). However, the mechanisms determining which phosphorylation pathway is activated remain unclear. Recent research suggests that the C‐terminal region of CK1δ plays a role in this regulation [39, 41]. Furthermore, the influence of CK1δ/ε on the CC extends beyond PER proteins. Indeed, these enzymes have also been shown to be able to phosphorylate BMAL1 and CRY proteins, suggesting their involvement in additional regulatory pathways [42]. The significant role played by CK1δ/ε is also highlighted by the discovered connection between mutations in these kinases or in their target phosphorylation sites and sleep disorders [42]. For instance, the specific R178C mutation on CK1ε has been found to lead to a shorter circadian period [39, 43], while the S662G mutation on PER2 prevents the proper phosphorylation by CK1δ/ε and leads to FASPS, which is characterized by earlier sleepiness and waking up times [44].

**Figure 3:**
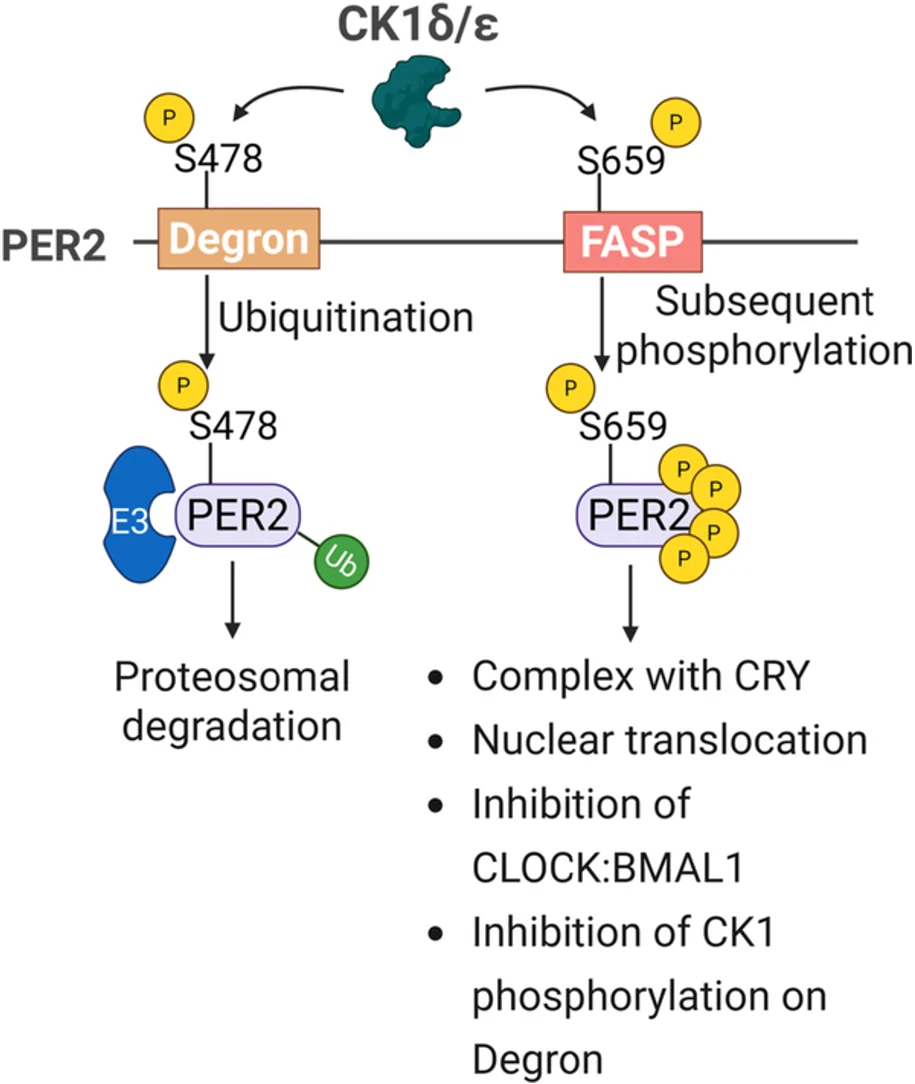
Phosphorylation of PER2 by CK1δ/ε on specific sites and subsequent effects on protein stability and function. Phosphorylation of Ser478 in the Degron region targets PER2 for ubiquitination and proteasomal degradation. Phosphorylation of Ser659 in the FASP region promotes further phosphorylation on nearby serine residues, leading to PER2 complex formation with CRY, nuclear translocation, and inhibition of the CLOCK:BMAL1 complex. Additionally, phosphorylation at Ser659 inhibits the phosphorylation of CK1δ/ε in the Degron region. Figure created with BioRender.com. [Color figure can be viewed at wileyonlinelibrary.com]

A major challenge in targeting CK1δ and CK1ε is designing selective inhibitors. Currently, all developed CK1 inhibitors act as competitive inhibitors at the ATP‐binding site, which, however, does not grant selectivity because of the highly conserved nature of the ATP‐binding domain across kinases. The selectivity issue is further amplified by the high degree of sequence homology between CK1δ and CK1ε. Indeed, these isoforms share 98% identity within the kinase domain, leaving minimal space for the development of highly specific inhibitors. Consequently, most CK1 inhibitors currently available act as pan‐CK1δ/ε inhibitors. For instance, compounds CKI‐7, IC‐261, D4476, LH846, and PF‐670462 (Figure 4 and Table 1) were identified among the first CK1δ/ε inhibitors. These compounds demonstrated the ability to lengthen the CC in *in vitro* phenotypic assay in a concentration‐dependent manner [18, 45, 46, 53, 54]. Additionally, phenotypic screens indicated that the majority of molecules identified as circadian period lengtheners were CK1δ/ε inhibitors [30, 55].

**Figure 4:**
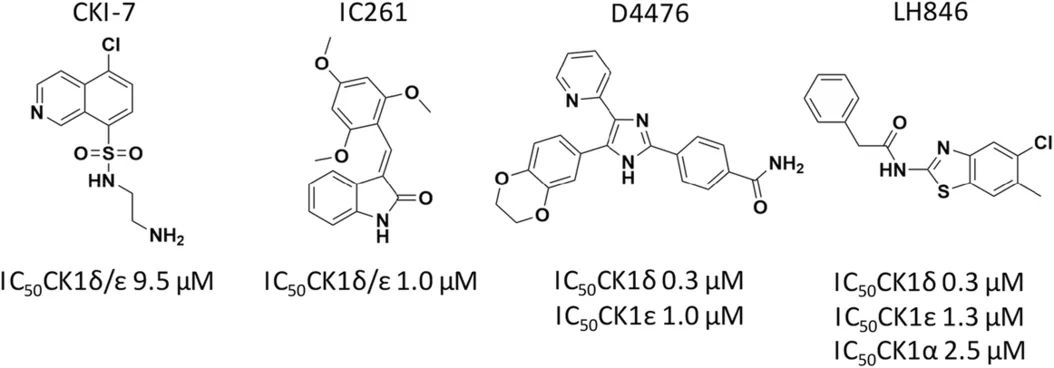
Structures and inhibitory activities, expressed as IC_50_ values [45, 46, 47, 48], of cited CK1δ/ε inhibitors.

**Table 1:** PF‐series of CK1δ/ε inhibitors with reported circadian effect *in vitro* and *in vivo*.

	PF‐670462	PF‐4800567	PF‐5006739	PF‐05251749
	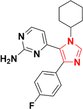	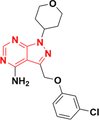	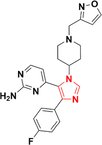	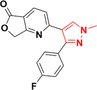
*In vitro* studies				
IC_50_ CK1δ [nM]	10.4	711	3.9	7.91
IC_50_ CK1ε [nM]	50.3	32	17.0	26.9
Selectivity IC_50_ CK1δ/IC_50_ CK1ε	0.21	22.22	0.23	0.29
EC_50_ CK1δ [nM]^a^	140.0	2 650.0	15.2	n/a
EC_50_ CK1ε [nM]^a^	550.0	130.0	83.0	n/a
CC effect *in vitro* b	Lengthening of the period	No effect reported	n/a	n/a
*In vivo* studies				
Adult male C57BL/6 J mice	−6.5 ± 0.23 h 32 mg/kg DD experiment	−0.51 ± 0.08 h 100 mg/kg DD experiment	−2.05 ± 0.31 h 17.8 mg/kg LD experiment	n/a
Adult male CD rats – DD experiment	−1.1 ± 0.3 h 10 mg/kg	n/a	−1.0 ± 0.6 h 10 mg/kg	n/a
4‐5 years old male Cynomolgus monkeys – LD experiment	−1.1 ± 0.3 h 10 mg/kg	n/a	−1.0 ± 0.6 h 10 mg/kg	n/a
Clinical trial	—	—	—	Early‐stage (good safety and tolerability)
Reference	[45, 49, 50]	[50]	[51]	[52]

Abbreviations: DD = constant darkness, LD = light‐dark cycle 12:12, n/a = not available.

^a^ CK1δ/ε whole‐cell assay using COS7 cells (African green monkey kidney fibroblast) and measured nuclear translocation of PER3GFP.

^b^ Circadian bioluminescence assay in mPer2dLuc Rat1 Cells.

Among identified CK1 inhibitors, PF670462 (see Table 1 for chemical structure) demonstrated promising *in vitro* activity in the nanomolar range (IC_50_ CK1δ = 10.4 nM, IC_50_ CK1ε = 50.3 nM). In functional whole‐cell assays, the compound confirmed its activity showing EC_50_ values of 140 nM and 550 nM for CK1δ and CK1ε, respectively. As expected, the EC_50_ values obtained in the whole cell assays were higher than the biochemical IC_50_ values on isolated enzymes. However, the difference in potency between CK1δ and CK1ε remained comparable across both assays, indicating a good correlation between enzymatic inhibition and cellular effects. PF670462 also showed a good selectivity against a panel of 45 kinases (its IC_50_ was < 0.3 μM only for Epidermal Growth Factor Receptor and p38 kinases), making it a promising candidate for *in vivo* studies. Experiments performed in not nocturnal species (rats and mice) and the diurnal species (cynomolgus monkey) showed that this compound was able to lengthen the circadian period in a dose‐dependent manner, and that the effect was influenced by the time of administration [45, 49]. PF760462 demonstrated a clear *in vivo* effect on the circadian period in both nocturnal and diurnal species, causing robust phase delays in the onset of locomotor activity (body clock and associated behaviors, like sleep and wake times, are shifted to a later time). Furthermore, preliminary data from the monkey study showing no signs of toxicity up to a dose of 100 mg/kg, making PF670462 particularly promising for further studies.

In mice with disrupted circadian rhythm, whether due to a genetic mutation (using Vipr2‐/‐ mice) or an environmental factor (using a constant light environment on WT mice), daily administration of PF670462 resulted in robust 24 h CC that lasted throughout treatment period [28]. Owing to the strong link between Alzheimer's disease (AD) and circadian rhythm disruption, as well as the overexpression of CK1δ enzyme in AD [56], PF670462 was studied in animal models of AD (3×Tg‐AD and APP‐PS1 mice models). Daily administration of PF670462 to 3×Tg‐AD mice improved memory function and restored normal circadian rhythm behavior [57]. Similarly, in APP‐PS1 mice, treatment with PF670462 lengthened the CC, improved cognition, and reduced Aβ brain load and plaque size in a dose‐dependent manner [58]. However, its high human liver microsomal clearance (HLM), resulting in a half‐life (t_1/2_) of less than 30 min, prevented its further development.

In the attempt to improve the pharmacokinetic properties and expand the chemical space, Wager et al. [51] produced a new set of compounds in which the imidazole scaffold was maintained and a piperidine moiety (instead of the cyclohexyl moiety in PF670462) bearing different substituents was investigated. The study led to the identification of PF5006739, which bears an isoxazole substituent (Table 1). This molecule showed an increased *in vitro* activity compared to PF670462, while maintaining excellent physical chemical properties and good selectivity. In addition, its HLM was significantly decreased (18.0 μL/min/mg, compared to 129.0 μL/min/mg of PF670462), and the passive membrane permeability was improved. *In vivo* studies confirmed the ability of PF5006739 to modulate circadian rhythm in a dose‐dependent manner in both mice and diurnal cynomolgus monkeys [51].

In parallel, Pfizer developed a new series of compounds featuring an imidazole ring instead of the pyrazole moiety present in PF670462 and PF5006739. This research led to the discovery of PF05251749 (Table 1, patented in 2012) [52]. This compound showed strong inhibitory potency against CK1δ/ε and high brain penetration. In pre‐clinical studies, it showed oral bioavailability of 46%–72% in rats and 44% in dogs, leading to its selection for further development [59]. PF05251749 successfully completed two clinical phase I trials (ClinicalTrials.gov Identifier: NCT02443740 and NCT02691702), showing good safety and tolerability. These findings advance the development of CK1δ/ε inhibitors as human therapeutics for Alzheimer's disease and circadian rhythm sleep disorders.

These findings establish a foundation for the continued development of CK1δ/ε inhibitors. Most of these molecules share a common scaffold consisting of a central nitrogen heterocycle linked to various hydrophobic groups. Furthermore, all identified compounds act as ATP‐competitive inhibitors, binding to the CK1δ‐ATP‐binding site in a consistent manner [60]. The inhibition mechanism of competitive inhibitors relies critically on a hydrogen bond interaction formed by Glu83, Leu84, and Leu85 in the hinge region of the ATP binding site, as well as interactions with the DFG motif of the protein via Asp149, Phe150, and Gly151. Additionally, a hydrogen bond with Lys38 and a hydrophobic interaction with Met82, the “gatekeeper residue” are common features, contributing also to selectivity against other kinases. The co‐crystallized structure of PF670462 with CK1δ exemplified this binding mode (Figure 5B). The amino‐pyrimidine moiety forms a hydrogen bond with Leu85, while the imidazole ring forms hydrogen bonds with Lys38 and Asp149. Additionally, the lipophilic fluorophenyl group occupies a hydrophobic pocket, interacting closely with Met82 and Met80 [61].

**Figure 5:**
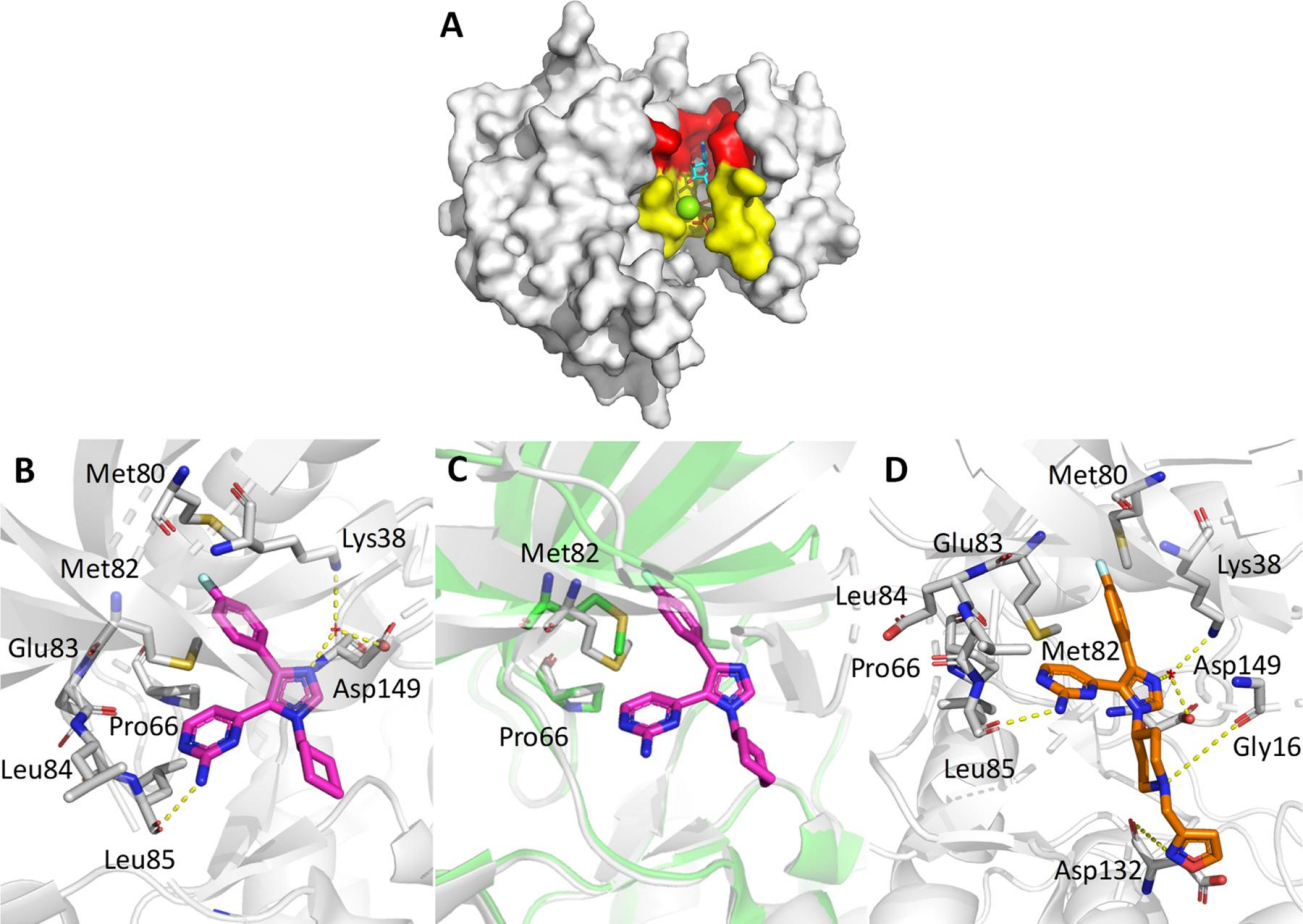
Binding pocket of CK1δ and mode of interaction of inhibitors. (A) Surface representation of CK1δ ATP‐binding pocket. PDB ID: 1CSN. The adenine‐binding region is highlighted in red, while the phosphate and ribose‐binding regions are highlighted in yellow. (B) Crystal structure of PF670462 (pink) binding to CK1δ (white). PDB ID: 3UZP. (C) Superimposition of apo‐CK1δ (green, PDB ID: 3UYS) and PF670462‐CK1δ (white). (D) Crystal structure of PF5006739 (orange) binding to CK1δ (white). PDB ID: 4TNS. Nitrogen atoms are depicted in blue, oxygen in red, fluorine in light blue, sulfur in yellow, and the H‐bonds with yellow dashed lines. Figure created with Pymol Molecular Graphics System 3.0. [Color figure can be viewed at wileyonlinelibrary.com]

The superimposition of the crystal structures of the apo‐CK1δ and the PF670462‐CK1δ complex revealed the key role of the residue Pro66 in granting PF670462 selectivity towards CK1δ (Figure 5C). Indeed, the presence of the small amino acid Pro66 induces a rotation of 180° of the Met82 residue and places it in the optimal position to interact with the inhibitor. This explained the good selectivity of PF670462 for CK1δ versus other kinases, in which the presence of bulkier moieties at this position hinders the rotation of the gatekeeper residue Met82, and also explains the inhibition of CK1ε, which retains the small Pro66 residue [61]. The crystal structure of the PF5006739/CK1δ complex (Figure 5D) showed that the binding mode of this analog overlaps with that of PF670462, but the expanded chemical space of PF5006739 allowed establishing additional interactions with Gly16 (via the piperidine amino group) and Asp132 (involving the nitrogen of the isoxazole ring). These additional interactions likely contribute to the stronger inhibitory activity of PF5006739 [51]. The crystal structure of the PF5006739/CK1δ complex (Figure 5D) showed that the binding mode of this analog overlaps with that of PF670462, but the expanded chemical space of PF5006739 allowed establishing additional interactions with Gly16 (via the piperidine amino group) and Asp132 (involving the nitrogen of the isoxazole ring). These additional interactions likely contribute to the stronger inhibitory activity of PF5006739.

To understand the specific functions of CK1δ and CK1ε in regulating the CC, and potentially identify a dominant player, Walton et al. developed the selective CK1ε inhibitor PF4800567 (see Table 1 for chemical structure), which exhibited a remarkable selectivity for CK1ε versus CK1δ higher than 20 fold [50]. This selectivity was maintained in whole cells, where the EC_50_ for CK1ε was 130.0 nM compared to 2650.0 nM for CK1δ. PF4800567 was found to be able to inhibit the nuclear translocation and degradation of PER. However, when tested in cells expressing the PER2 reporter *Per2*‐d*Luc* (circadian bioluminescence assay), PF4800567 had only a minimal effect on the period length, even at high concentrations (up to 30 μM). This result was in sharp contrast with those obtained with the pan‐CK1δ/ε inhibitor PF670462, which was able to significantly extend the period at a concentration of only 1 μM. This difference was also observed in *in vivo* studies. When tested in mice, at concentrations dosed based on their respective EC_50_ values in cells, PF670462 significantly delayed the active period while PF4800567 caused only a small delay, which indeed might be related to a weak CK1δ inhibition at such a high dose [50]. These findings led to the assumption that CK1ε is not the predominant mediator of CC compared to CK1δ. Additionally, the study by Walton et al. highlighted another important point: the significant *in vivo* effect exerted by PF670462 occurred at a concentration that had only a very small effect on Rat1 cells *in vitro*. This may indicate that in animals, CC is much more sensitive to CK1δ/ε inhibition compared to isolated cells [50].

The key structural motives leading to PF4800567 selectivity towards CK1ε were investigated by Long et al. by X‐ray crystallography, which revealed significant insights into its binding mode [62]. They co‐crystallized the inhibitor with both CK1δ and CK1ε, allowing a detailed comparison of the binding modes (Figure 6). The analysis of the two complexes revealed a similar interaction pattern of PF670462 with CK1δ and CK1ε: the amino‐pyrimidine group interacts with the hinge region of the pocket through Glu83 and Leu85, while the meta‐chlorophenyl group resides in a hydrophobic pocket and interacts with Met80/Met82 residues, plus rotates towards Pro66. However, a key difference emerged in the DFG motif. The crystal structure of PF4800567/CK1ε complex displayed a “DFG‐out” conformation, in which the flipped Phe150 residue engages strong hydrophobic interactions with the meta‐chlorophenyl moiety of the inhibitor. In contrast, the PF4800567/CK1δ complex exhibited a “DFG‐in” configuration, in which Phe150 remains positioned within the pocket. This difference in the orientation of the DFG motif was suggested to be pivotal for the selectivity of PF4800567 towards CK1ε (Figure 6B) [62].

**Figure 6:**
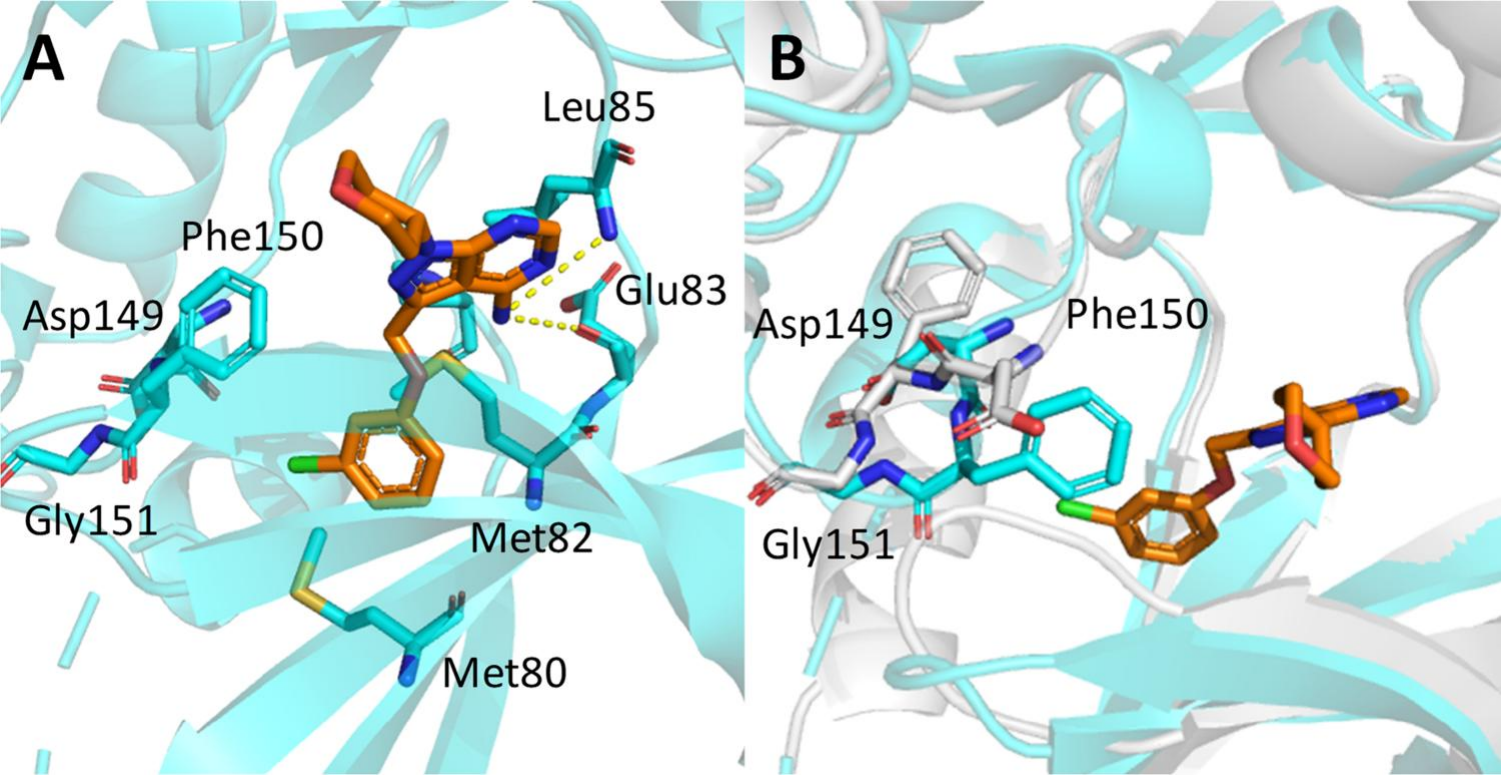
Mode of interaction of PF4800567 with CK1δ and CK1ε. (A) Crystal structure of PF4800567 (orange) bound to CK1ε (light blue). PDB ID: 4HNI. (B) Superimposition of PF4800567‐CK1ε (light blue) and PF4800567‐CK1δ (white, PDB code: 4HNF). Nitrogen atoms are depicted in blue, oxygen in red, fluorine in light blue, sulfur in yellow, and the H‐bond with yellow dashed lines. Figure created with Pymol Molecular Graphics System 3.0. [Color figure can be viewed at wileyonlinelibrary.com]

To date, there are no covalent (or allosteric) inhibitors of CK1δ/ε. The absence of cysteine residues in the active site of CK1δ/ε hinders the development of covalent inhibitors. While the catalytic residue Lys38 could potentially be exploited for covalent inhibition, significant challenges remain, and irreversible CK1δ inhibitors have yet to be developed [63].

Casein kinase 1 alpha (CK1α) has also emerged as a potential target for modulating CC. Hirota et al. identified longdaysin (Figure 7) as a molecule able to lengthen the circadian rhythm in a cell‐based assay, using human osteosarcoma U2OS cells expressing the *Bmal1*‐d*Luc reporter* [64]. Subsequent studies revealed that longdaysin inhibited CK1δ and CK1α and, to a lesser extent, Extracellular Signal‐Regulated Kinase 2 (ERK2) and Cyclin‐Dependent Kinase 7 (CDK7). The cellular functional potency was also determined by PER1 degradation assays in HEK293T cells, yielding EC_50_ values of 9.7 μM for CK1δ and 9.2 μM for CK1α. To dissect the specific role of CK1α, the authors investigated the effects of longdaysin in cells prepared from CK1δ‐deficient mice harboring the mPer2Luc reporter. The study demonstrated that longdaysin was still able to lengthen the circadian period even in the absence of the CK1δ isoform. Additionally, it was demonstrated that CK1α binds to PER1 protein and regulates its stability [64]. Further experiments on a *Drosophila* model corroborated these findings [65] by confirming the ability of CK1α to phosphorylate PER protein; this phosphorylation promoted the interaction between PER and Doubletime (the *Drosophila* counterpart of human CK1δ/ε), with consequent PER phosphorylation and degradation. These results align with the observed effects of longdaysin. Indeed, CK1α inhibition by longdaysin leads to an increased PER protein stability and lengthens the circadian period [65].

**Figure 7:**
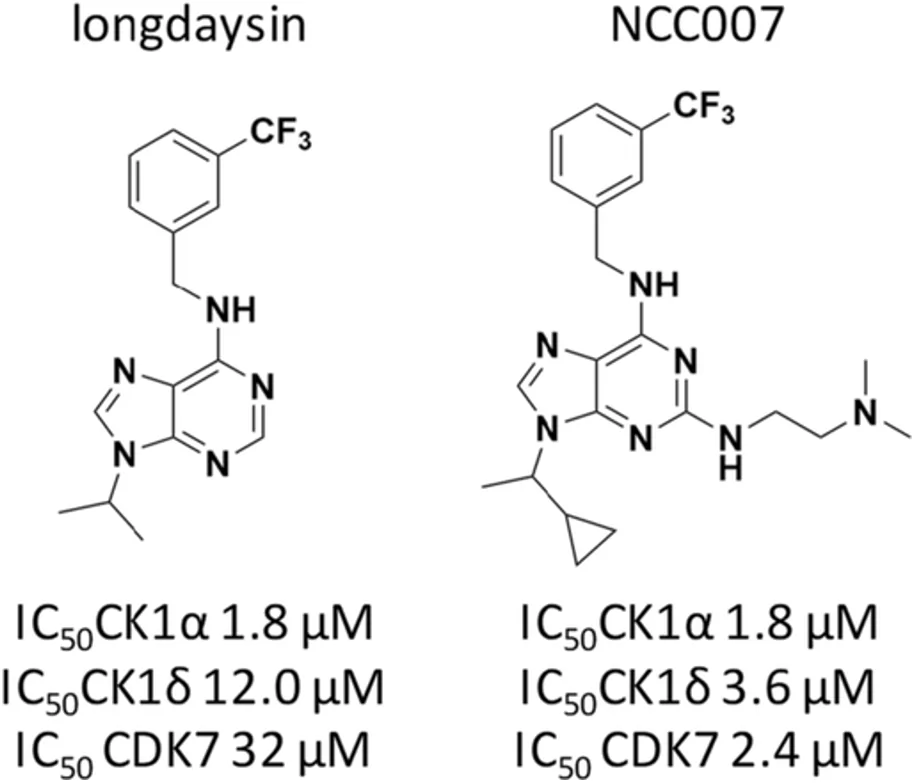
Structures and inhibitory activities, expressed as IC_50_ values [27, 64], of cited CK1α/δ inhibitors.

In 2019, another study explored the structure‐activity relationship (SAR) of longdaysin, leading to the discovery of its derivative NCC007 (Figure 7). NCC007 exhibited stronger inhibitory potency against CK1δ, CK1α, and CDK7. The improved effect was also confirmed in *Bmal1‐dLuc* U2OS cell assay in which NCC007 was able to lengthen the circadian period by 5 h when tested at 0.32 μM. Interestingly, the *in vivo* effects of both longdaysin and NCC007 in C57BL/6 J background male mice (8–week‐old) were significantly less pronounced. Cerebrospinal infusion of 5 mM longdaysin only lengthened the CC period by 0.1 h, and NCC007 showed a similar effect at the same concentration [27]. This discrepancy between *in vitro* and *in vivo* findings highlights the complexity of translating cellular observations to a living organism. Further research is needed to understand the factors contributing to such differences and optimize the efficacy of CK1α inhibitors for modulation of the CC *in vivo*.

### 1.6 Exploring the Use of CK1 Inhibitors in Chronophotopharmacology

Chronophotopharmacology is an emerging field that combines pharmacology with light‐based control to precisely modulate circadian rhythms in a spatiotemporal region [66]. It involves the use of light‐responsive molecules to modulate the biological clock and treat CC misalignment. One strategy to create photoswitchable ligands is photocaging, a chemical approach in which a photosensitive protecting group is attached to a biologically active molecule. Upon exposure to light at a specific wavelength, the photocage is cleaved and the active molecule is released. Kolarski et al. applied this strategy to the CK1α/δ inhibitor longdaysin, obtaining DK359 (Figure 8A), in which the photocleavable nitroveratryloxycarbonyl group (NVOC) has been introduced on the exocyclic amine at position‐6 of the purine scaffold, which is essential for the activity of the compound. Irradiation at 365 or 400 nm leads to the release of longdaysin which can then exert its action. Investigations in human cells, mouse tissues, and *in vivo* in zebrafish have shown that the lengthening of the circadian period was irradiation‐duration‐dependent [67].

**Figure 8:**
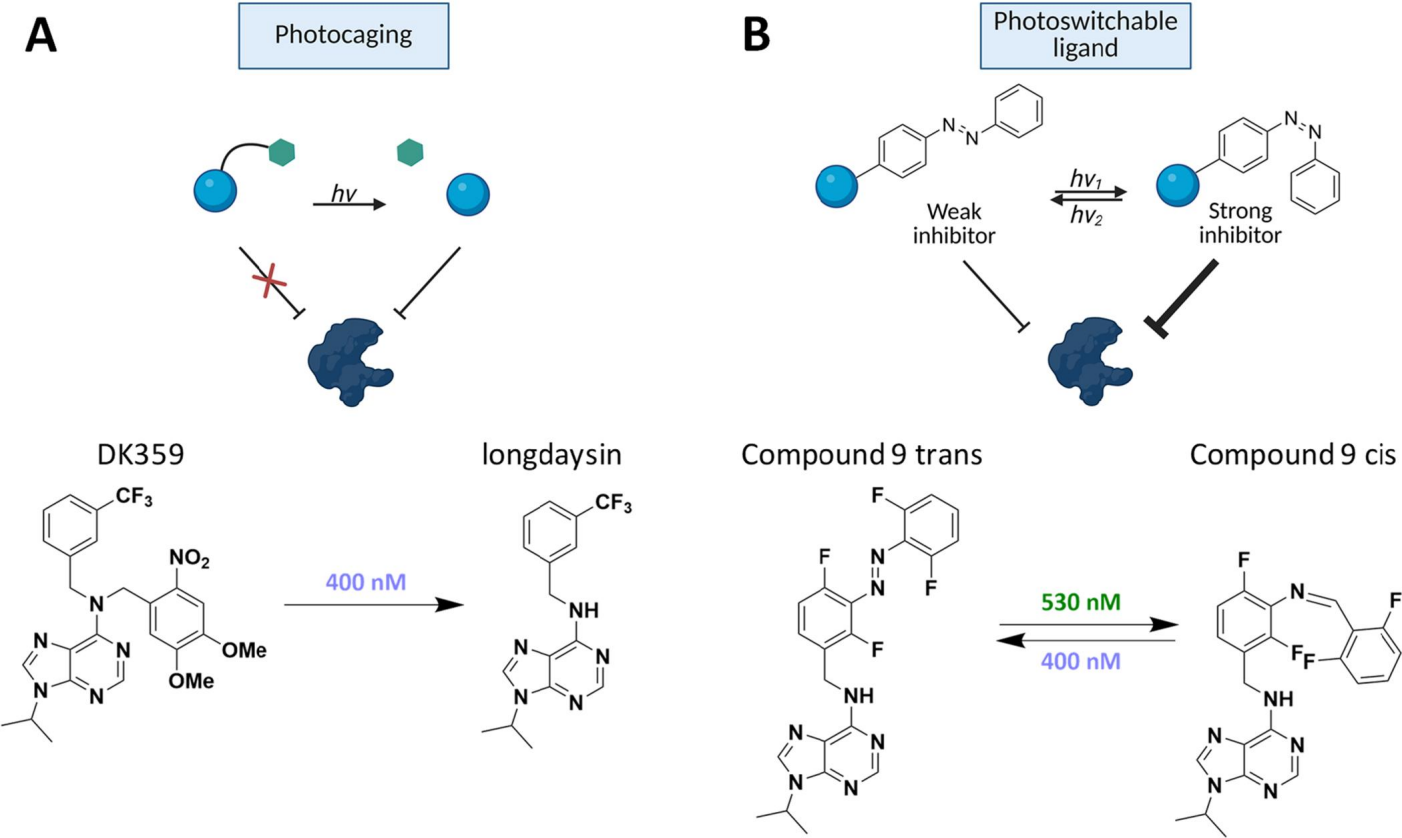
General principle of two chemical approaches for generating photo‐switchable molecules to be used in chronophotopharmacology: (A) photocaging and (B) incorporation of photo‐isomerizable molecules, exemplified by attempts with longdaysin. Figure created with Pymol Molecular Graphics System 3.0. [Color figure can be viewed at wileyonlinelibrary.com]

Another strategy foresees the incorporation of photo‐isomerizable molecules, such as azobenzene, into the ligand structure. This leads to an inactive, or slightly active molecule, that can be activated by light‐induced conformational changes in a reversible manner. This approach has been applied to both longdaysin and the CK1δ/ε inhibitor LH846 [68, 69, 70]. It is important to note that compound 9 (Figure 8B), obtained from longdaysin, upon in situ photoisomerization with visible light, could modulate CK1 activity in U2OS cells for multiple days. While *in vivo* confirmations are still missed, this approach holds significant promise for further chronophotopharmacology research and development.

### 1.7 Casein Kinase 2

Casein kinase 2 (CK2) is a ubiquitous serine/threonine kinase composed of four subunits: two catalytic subunits (α and α', highly similar at 90% sequence identity) and two regulatory β subunits (Figure 9A) [71]. This enzyme is involved in various cellular pathways, including cell growth, apoptosis, and DNA damage repair. For these reasons, it is widely investigated as a target for cancer therapy [72]. CK2 also emerges as a key conductor within the CC. It phosphorylates PER2, regulates its accumulation within the cell nucleus [73], and collaborates with CK1ε to promote PER2 degradation [74]. Additionally, CK2 phosphorylates BMAL1, leading to its nuclear accumulation as well. Such phosphorylation of BMAL1 is essential for its heterodimerization with CLOCK [7].

**Figure 9:**
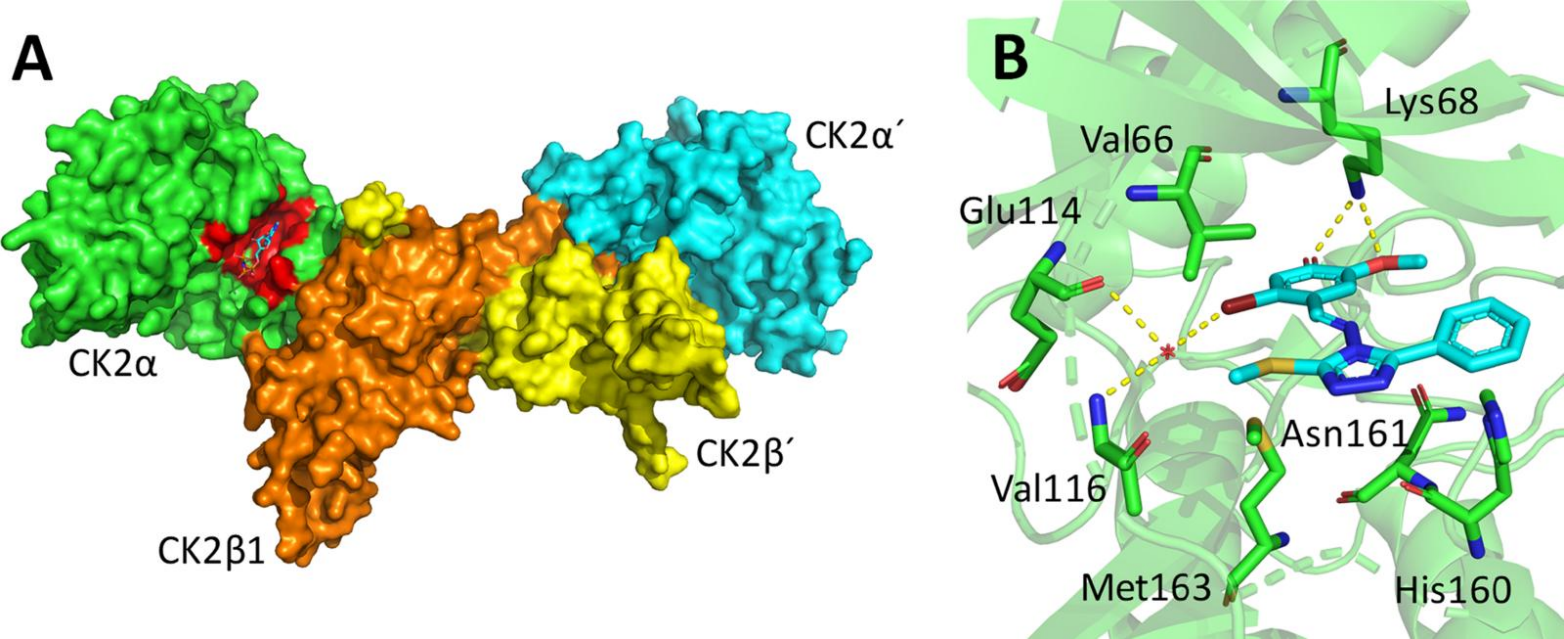
Binding pocket of CK2 and mode of interaction of inhibitor GO289. (A) Surface representation of the CK2 protein complex. The ATP‐binding pocket in the CK2α chain is highlighted in red. PDB ID: 1JWH. (B) Crystal structure of GO289 (light blue) binding to CK2α (green). PDB ID: 6A1C. Nitrogen atoms are depicted in blue, oxygen in red, bromine in dark red, sulfur in yellow, and the H‐bond with yellow dashed lines. Figure created with Pymol Molecular Graphics System 3.0. [Color figure can be viewed at wileyonlinelibrary.com]

Further evidence supporting the role of CK2 in the CC was achieved with the inhibitor 2‐dimethylamino‐4,5,6,7‐tetrabromo‐benzimidazole (DMAT, Figure 10) that was able to slightly extend the circadian period in mouse NIH3T3 cells with *Bmal1‐*d*luc* reporter system [74]. However, the lack of selectivity of this molecule towards other kinases, Protein Kinase D1 (PKD1) and Proviral Integration site for Moloney murine leukemia virus (PIM) family kinases, hindered the straightforward interpretation of the results [75].

**Figure 10:**
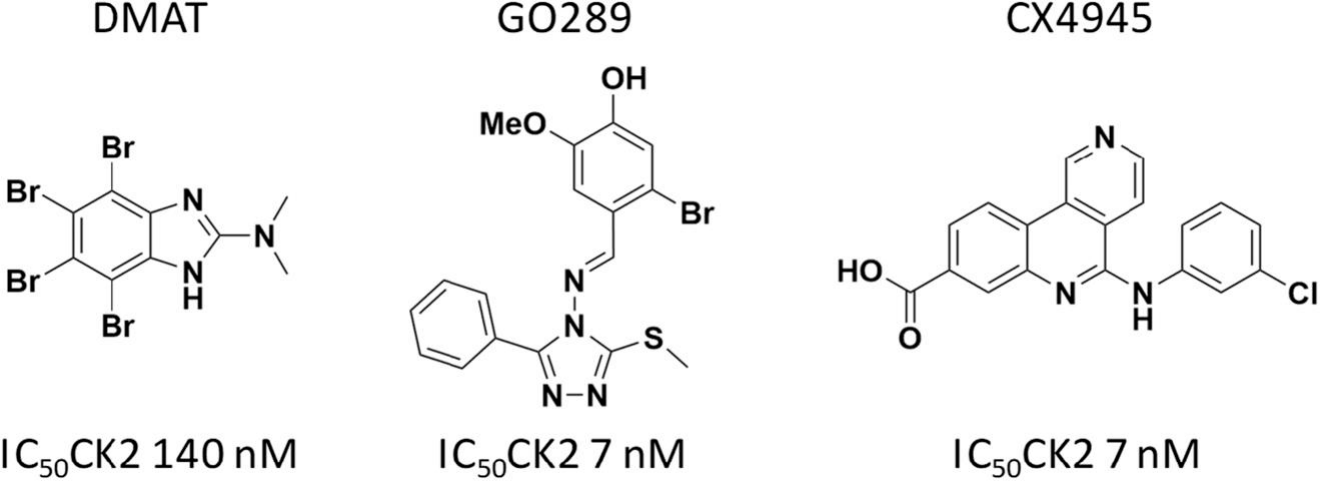
Structure and inhibitory activities, expressed as IC_50_ values [32, 74], of cited CK2 inhibitors.

A significant step forward came in 2019 with the discovery of GO289 (Figure 10) by Oshima et al. [32] Using an affinity‐based proteomic approach in human U2OS cells with the *Bmal1‐*d*Luc* reporter system [76], this guaiacol derivative was identified as a new potent and selective ATP‐competitive CK2 inhibitor. It was able to extend the circadian period in *Bmal1*‐d*Luc* and *Per2*‐d*Luc* reporter cell lines. GO289 displayed only a weak inhibitory activity against CK1δ, CK1α, and other kinases commonly targeted by CK2 inhibitors, including dual‐specificity tyrosine phosphorylation‐regulated kinase (DYRK), homeodomain‐interacting protein kinase (HIPK), and PIM family kinases. This remarkable selectivity, quantified as being > 1000 times higher for CK2 than for the next closest off‐target kinase (PIM2), made GO289 a valuable tool for precisely dissecting the specific role of CK2 in regulating the CC within cells. However, studies investigating the effects of GO289 *in vivo* are currently lacking. This is a critical gap, particularly given the observed discrepancies between *in vitro* and *in vivo* activity seen with CK1 inhibitors. Therefore, exploring the efficacy of GO289 in animal models will be crucial to truly assess its potential in treating circadian rhythm disorders.

Further, GO289 was crystallized in complex with human CK2 α‐subunit. The crystal structure revealed a significant number of hydrophobic and hydrophilic interactions within the ATP‐binding site (Figure 9B). The hydroxy and methoxy groups of the guaiacol moiety formed hydrogen bonds with Lys68, while the phenyl ring was engaged in a hydrophobic interaction with the imidazole ring of His160, and the methylthioether group interacted with Val66 and Met163. Additionally, the bromine substituent on the guaiacol moiety formed a water‐mediated hydrogen interaction with Glu114 and Val116, which are located in the hinge region of CK2. This region, where many kinase inhibitors form crucial hydrogen bonds, is highly conserved among kinases. The absence of direct interactions within this region in GO289 may contribute to its higher selectivity compared to other kinase inhibitors [32].

The well‐known CK2 inhibitor CX‐4945 (Figure 10) was also found to be able to lengthen the CC in Bmal1‐d*Luc* U2OS cells [32]. This finding is particularly interesting because this compound is currently (2025) undergoing clinical trials for its antitumor activity (ClinicalTrials.gov identifier NCT02128282), and it has already shown good tolerability and pharmacokinetic properties in humans [77].

### 1.8 Glycogen Synthase Kinase 3β

GSK3β is a ubiquitous serine/threonine kinase involved in various cytosolic and nuclear functions [78] that possesses many core‐clock proteins as substrates. Phosphorylation of REV‐ERBα by GSK3β stabilizes the protein, while phosphorylation of BMAL1 and CLOCK targets them for proteasomal degradation [11, 12]. Furthermore, GSK3β is involved in the nuclear translocation of PER2 and in the stabilization of CRY [79].

Despite the clear involvement of GSK3β in the CC, the effects of its inhibition on circadian rhythm are complex and not yet fully understood. Although the lithium, known for its ability to inhibit GSK3β, has been shown to lengthen the circadian period in a dose‐dependent manner *in vitro* and *in vivo* [19, 80, 81], the GSK3β inhibitors CHIR99021 (laduviglusib) [82] and 1‐azakenpaullone [83] (Figure 11) have provided contradictory results. When tested in *Bmal1*‐d*luc* U2OS cells, these inhibitors were found to be able to shorten the circadian period [76]. Similarly, the inhibitor SB216763 [84] (Figure 11) shortened the circadian period when tested in *Bmal1*‐d*luc* Rat1 fibroblasts [85]. Furthermore, siRNA‐mediated knockdown of GSK3β in *Bmal1*‐d*luc* U2OS cells shortened the circadian period, further supporting the idea that GSK3β inhibition can decrease the period length [76]. Such discrepancies may be attributed to the lack of selectivity of lithium [86, 87], which may also affect other pathways that influence circadian rhythms [88, 89].

**Figure 11:**
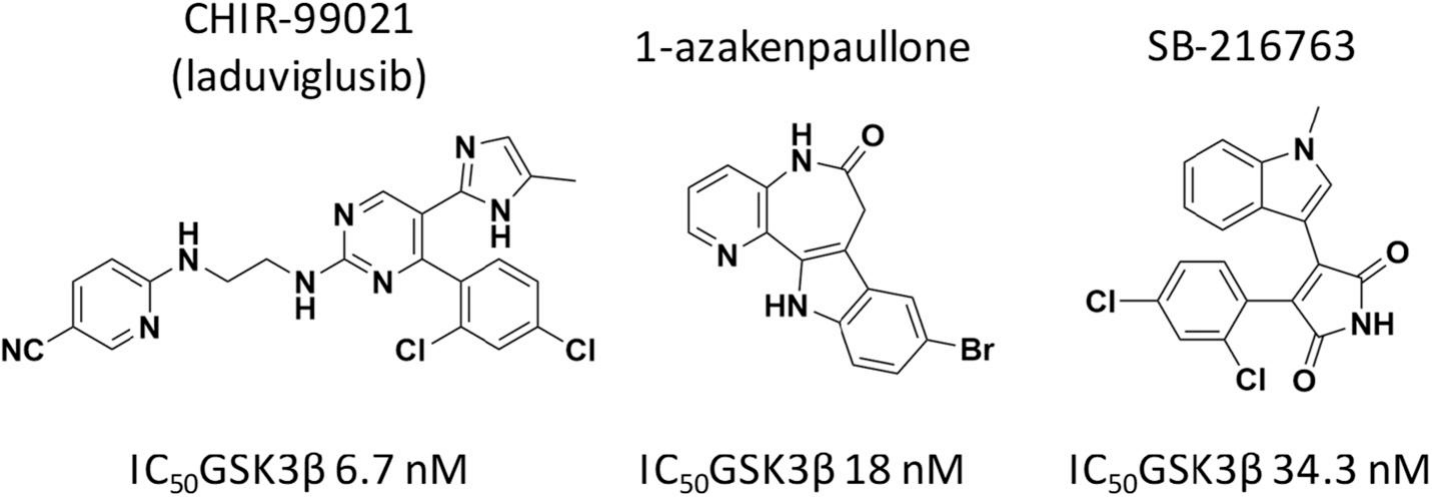
Structures and inhibitory potencies [82, 83, 84] of GSK3β inhibitors.

Additionally, recent evidence has highlighted potential off‐target effects on other kinases that may be inhibited by CHIR99021 and SB216763 and that may confound the interpretation of the results. To gain a clearer understanding of the role of GSK3β in circadian regulation, further studies with highly selective GSK3β inhibitors are needed [90]. By specifically targeting GSK3β, researchers could elucidate the precise mechanisms by which this kinase influences circadian rhythms and explore its potential as a therapeutic target for circadian‐related disorders.

### 1.9 Cyclin Dependent Kinases

Cyclin‐dependent kinases (CDKs) are heterodimeric serine/threonine kinases implicated in numerous processes in the cell, the most prominent being the cell cycle and the transcriptional cycle. Twenty isoforms have been identified in humans. While they are primarily studied as anticancer targets, their involvement in circadian rhythm and their coupling with the cell cycle have attracted increasing attention [91]. Initial screening by Hirota et al. identified a link between CDKs and circadian clock, as the CDK/GSK3β inhibitors indirubin‐3'‐oxime and kenpaullone demonstrated the ability to shortening the circadian period in *Bmal1*‐d*luc* U2OS cells.

Further investigations into the role of specific CDKs revealed that CDK5 may play a major role in circadian regulation. This was initially supported by studies with known CDKs inhibitors: roscovitine [92] (Figure 12), a CDK2/CDK5 inhibitor, lengthened the circadian period, while the CDK1/CDK2 inhibitor NU6102 [93] (Figure 12) did not have an effect on the CC, suggesting a crucial role for CDK5 [76]. Molecular studies subsequently showed that CDK5 can directly phosphorylate the CLOCK protein [8], regulating its stability and cellular distribution. Additionally, CDK5 phosphorylates also the PER2 protein, promoting its nuclear translocation and stabilization [10]; interestingly, CDK5 in cells phosphorylates and inhibits CK1δ activity [96]. This latter point is crucial, as the *in vitro* lengthening of the circadian period by roscovitine contrasts with the *in vivo* result where knockdown of *Cdk5* in the suprachiasmatic nuclei in mice led to a shortened free‐running period [10]. This discrepancy may be attributed to the lack of a poor selectivity of roscovitine, which can also inhibit CK1δ, potentially obscuring the true CDK5 effect in the cell‐based assay. [30].

**Figure 12:**
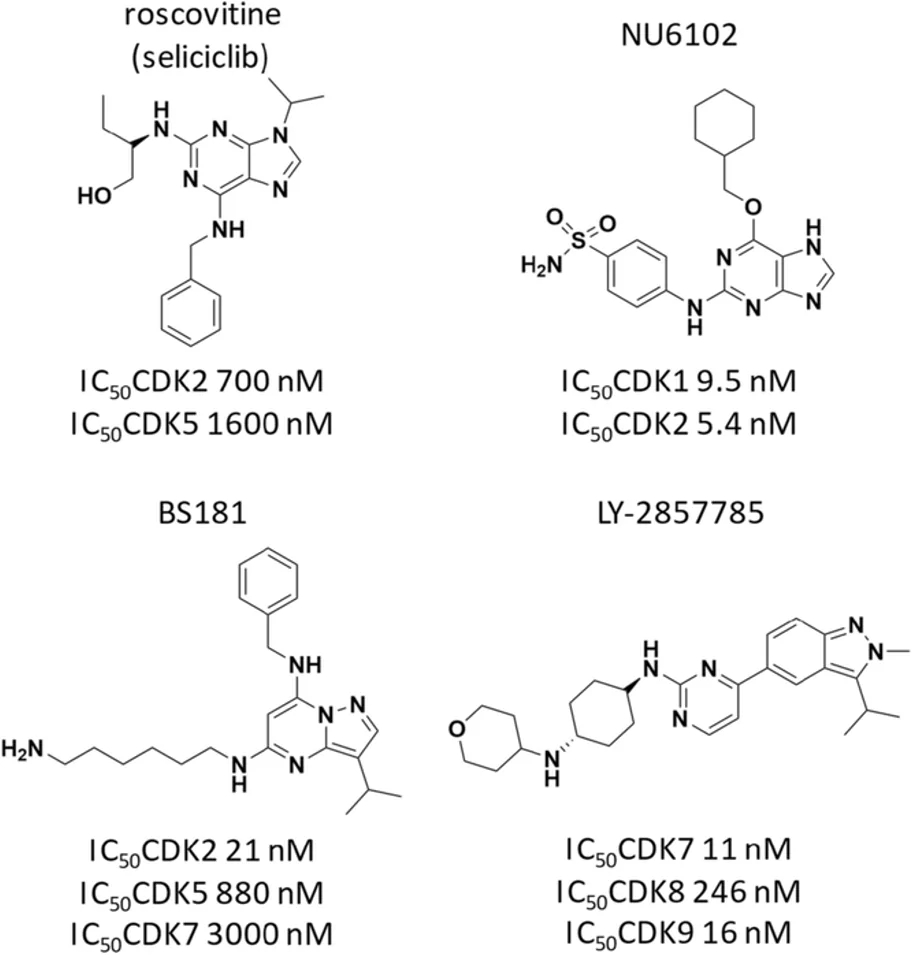
Structures and inhibitory activities, expressed as IC_50_ values [92, 93, 94, 95], of cited CDKs inhibitors.

CDK9 has also emerged as an important player in circadian regulation. Ou et al. screened 17 known CDK inhibitors in *mPer2*‐d*Luc* MEF cells and identified LY2857785 [94] and BS181 [95] (Figure 12) as compounds able to decrease *mPer2*‐d*Luc* amplitude [97]. To dissect the specific effect for each CDK enzyme, the authors alternately knocked down *Cdk2*, *Cdk5*, *Cdk7*, *Cdk8*, *Cdk9* genes using siRNAs in *mper2‐dluc* mefs. Among those, only the *Cdk9* knockdown led to a clear reduction of the luminescence activity. Furthermore, *Cdk9*‐deficient mice exhibited increased respiratory exchange ratio and reduced locomotor activity during the dark phase. *In vitro* and *in vivo* evidence revealed that CDK9 acts by phosphorylating and suppressing REV‐ERBα levels, thereby affecting its interaction with the RORE of Bmal1 [97]. Thus, LY2857785 and its analogs may hold promise as potential therapeutic agents for circadian disorders.

### 1.10 AMPK‐Related Kinase Family

AMP‐activated protein kinase (AMPK) is a heterotrimeric protein kinase that plays a crucial role in maintaining cellular energy homeostasis and metabolism. Given the strong connection between metabolism and circadian rhythms [24], it is likely that AMPK also plays a role in the CC. AMPK phosphorylates CRY, which leads to its degradation. Furthermore, AMPK by phosphorylating and activating CK1ε indirectly destabilizes PER2 [98, 99, 100]. Metformin, a commonly used drug for type 2 diabetes, is able to activate AMPK and has been shown to shorten the period length in rat‐1 fibroblasts expressing *Per2*‐d*Luc*, and to reduce PER2 levels through a CK1ε‐mediated pathway [101]. However, attempts to inhibit AMPK using dorsomorphin, a known inhibitor of the AMPK pathway were complicated by its lack of selectivity (i.e., dorsomorphin inhibits ALK2, ALK3, ALK6, other than AMPK), hindering a clear interpretation of its effects on the CC cause by AMPK inhibition [102, 103]. The absence of highly selective AMPK inhibitors further hampers our understanding of the potential role of this kinase in circadian regulation.

Within the AMPK superfamily, the three salt‐inducible kinases (SIKs): SIK1, SIK2, and SIK3, have attracted attention for their role in the CC [104]. SIK1 plays a role in suppressing the effects of light on the CC [105, 106] and *Sik1*‐knockdown in mouse SCN, the principal circadian clock that coordinates daily behavior in response to environmental cues, led to an increased sensitivity to light and phase shifts, resembling jet lag behavior [106]. SIK3 is involved in the destabilization of PER2, promoting its phosphorylation and degradation. *Sik3‐/‐* mice exhibited phase delays, longer circadian periods, impaired light‐entrainment, and reduced amplitude in locomotor activity rhythms, suggesting a role for SIK3 in coordinating circadian rhythms and photic signaling [107]. The SIK inhibitors YKL‐05‐099 [108] and ARN‐3236 [109] (Figure 13) have been shown to perturb the cellular clock mechanism and decrease amplitude in U2OS *Bmal1*‐d*luc* cells [111]. Additionally, the administration of pan‐SIK inhibitor HG‐9‐91‐01 [110] (Figure 13) to sleep‐deprived mice has been shown to reduce the phosphorylation of Synaptic Sleep‐Need‐Index PhosPhoproteins (SNIPPs), proteins in synapses whose phosphorylation increases as the need for sleep [112]. While these findings suggest the potential of SIK inhibitors for treating circadian‐related disorders, further *in vivo* studies are essential to clarify their systemic effects.

**Figure 13:**
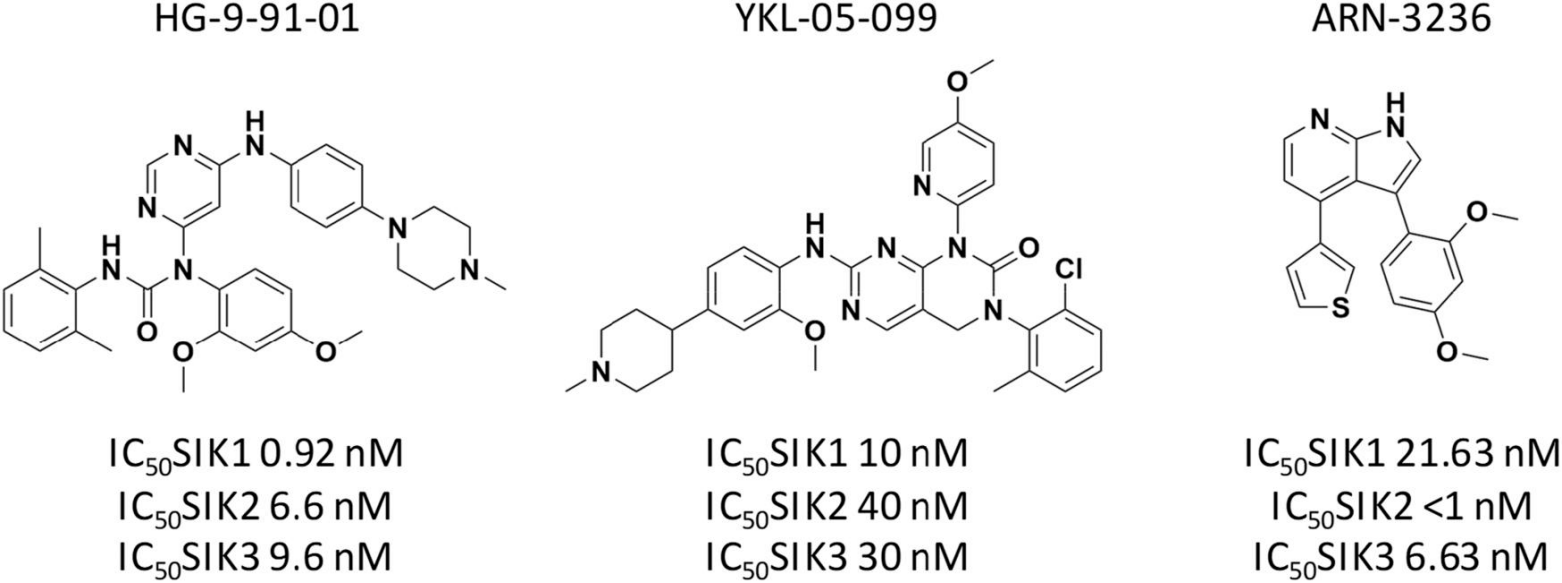
Structures and inhibitory activities, expressed as IC_50_ values [108, 109, 110], of cited SIKs inhibitors.

### 1.11 Ca^2+^/Calmodulin‐Dependent Protein Kinase II (CaMKII)

CaMKII was initially discovered to play an important role in the photic input pathway and rhythmic gene expression of clock genes [105, 113]. Further studies have demonstrated its crucial role in the cellular clock system, as it directly phosphorylates CLOCK and BMAL, enhancing their dimerization and E‐box‐dependent gene expression [85, 114]. The genetic ablation of CaMKII in C57BL/6 mice resulted in decoupling of morning and evening activity rhythms, sometimes leading to arrhythmicity [114]. The CaMKII inhibitor KN93 (Figure 14) shortened the circadian period in Rat‐1 fibroblasts expressing *Bmal1*‐d*Luc* and decreased the amplitude of the signal [85]. In accordance, the neurogenic small molecule ISX‐9 (Figure 14) increased the circadian amplitude of oscillation and improved sleep homeostasis in mice by increasing CaMKII expression [116]. However, it is important to note that KN93 and ISX‐9 are not selective. KN93 inhibits the interaction of the Ca^2+^/CaM complex with CaMKII, potentially affecting other Ca^2+^/CaM‐dependent enzymes [117], while ISX‐9 is a neurogenic molecule with broader effects. To elucidate the effect of inhibiting CaMKII, the use of a new generation of more potent and selective inhibitors, such as those proposed by Allosteros Therapeutics (e.g., AS105, Figure 14), is essential. These ATP‐competitive inhibitors are more potent and selective than KN93 and could help to clarify the specific role of CaMKII in the CC [115].

**Figure 14:**
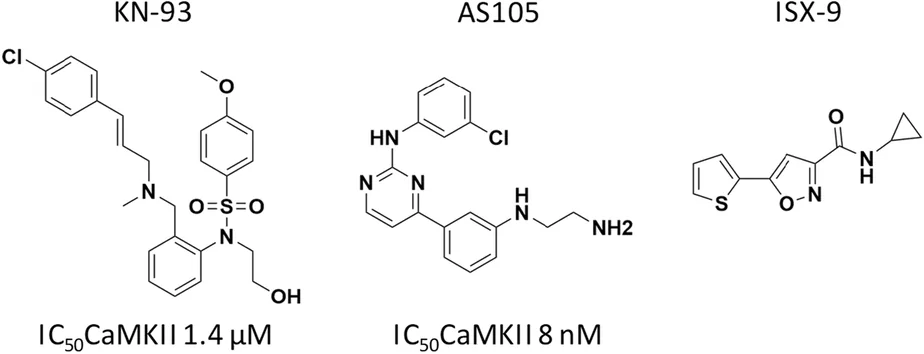
Structures and inhibitory activity, expressed as IC_50_ values [114, 115], of cited CaMKII inhibitors, and chemical structure of the neurogenic molecule ISX‐9.

### 1.12 Other Kinases Involved in the Circadian Clock

Other kinases have also been implicated in circadian regulation. Due to the complexity of the CC, the number of kinases involved is likely greater than that discussed in this review, but the roles of many of them and the impact of their inhibition remain unclear. For instance, tyrosine kinases ABL1, ABL2, and BCR have been suggested to play a role in the CC, on the basis of the ability of the BCR‐ABL inhibitors nilotinib and imatinib (Figure 15) to shorten the circadian period in U2OS‐*Bmal1*‐d*Luc* cells [118]. The mitogen‐activated protein kinases (MAPKs), including JNK and p38 MAPK, have also been suggested to be implicated in mammalian circadian rhythms [120]. The inhibitors of these kinases SP600125, SB203580, and SB202190 (Figure 15) have been shown to lengthen the circadian period *in vitro* [119, 121, 122]. However, it is important to note that these inhibitors often target multiple kinases, among which is CK1δ, making it difficult to definitively attribute their effects on the CC to a specific target [30]. Further research is needed to fully elucidate the roles of other kinases in circadian regulation and to develop more selective inhibitors as probes for proving specific functions.

**Figure 15:**
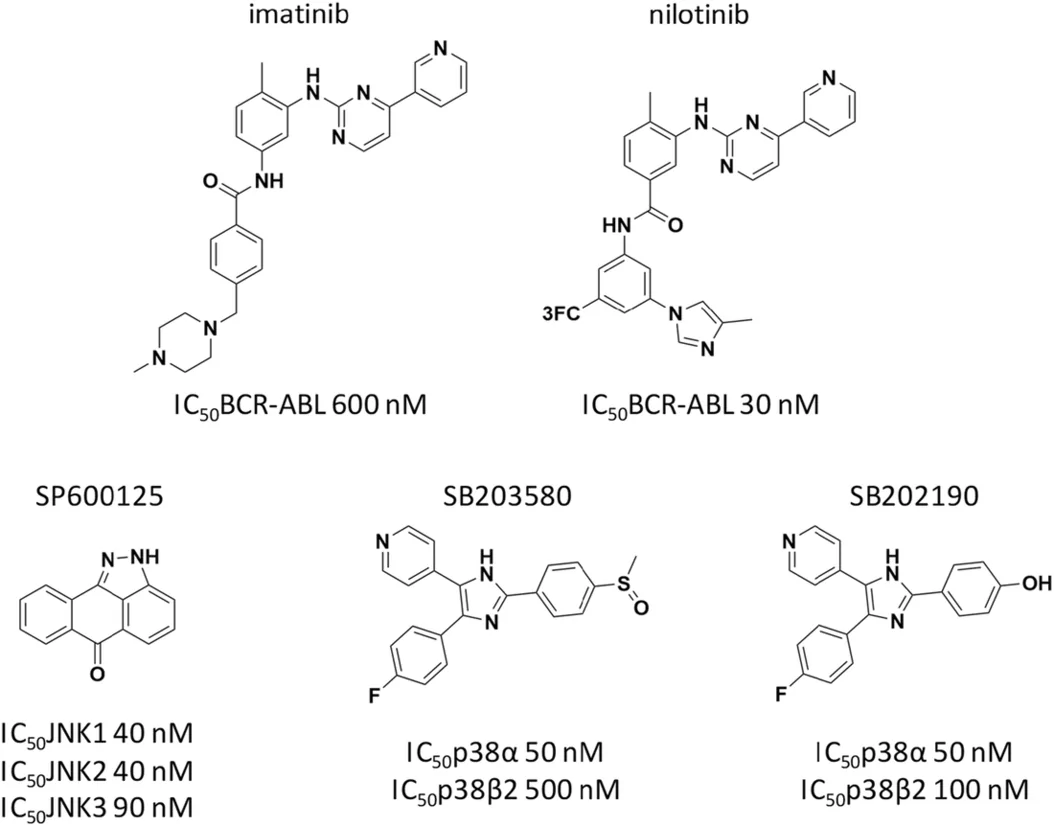
Structures and inhibitory activities, expressed as IC_50_ values [118, 119], of cited BCR‐ABL and MAPK inhibitors.

## 2 Conclusions and Outlook

In conclusion, kinases have emerged as fundamental components of the circadian clock (CC), and their inhibition offers a valuable strategy to modulate circadian rhythms, making them attractive drug targets for circadian‐related disorders. Within kinases, CK1δ/ε and CK2 have emerged as particularly promising targets for addressing CC‐related disorders due to their crucial roles in the transcription‐translation feedback loops and the successful development of potent small‐molecule inhibitors. The progression of compound PF‐05251749 into early‐stage clinical trials represents not only a significant milestone but also a hopeful leap towards effective therapeutic applications for circadian rhythm sleep disorders in humans.

However, for other kinases, such as GSK3β, SIKs, and CaMKII, the scenario is more debated. Indeed, despite an established general involvement in the CC, their precise roles and potential therapeutic benefits of their inhibition remain unclear. This uncertainty largely stems from the lack of selectivity of currently available inhibitors, which makes it difficult to assign observed effects to a specific kinase. Therefore, further studies with highly selective inhibitors are essential to fully elucidate the precise roles of those kinases in circadian regulation and validate them as molecular targets for CC related disorders.

A promising emerging strategy in CC modulation is chronophotopharmacology. Photoswitchable inhibitors against kinases, such as derivatives of longdaysin and LH846, have been synthesized and assayed. This approach offers a valuable strategy for the precise and reversible modulation of circadian rhythms with spatiotemporal control.

Finally and importantly, the literature analysis has highlighted a significant discrepancy between the effects observed *in vitro* and those seen *in vivo*. For instance, while longdaysin and NC007 were able to exert a promising *in vitro* activity, their effectiveness *in vivo* was limited. This underscores the importance of conducting *in vivo* studies to validate the outcomes from cell‐based assays and to better understand the complex interplay of factors influencing circadian rhythms in whole organisms. Such *in vivo* validation is crucial for translating the promising *in vitro* activity of kinase inhibitors into effective therapeutic strategies for circadian‐related disorders.

## Conflicts of Interest

The authors declare no conflicts of interest.

## Data Availability

The data that support the findings of this study are available from the corresponding author upon reasonable request.
